# The Cytolytic Amphipathic β(2,2)-Amino Acid LTX-401 Induces DAMP Release in Melanoma Cells and Causes Complete Regression of B16 Melanoma

**DOI:** 10.1371/journal.pone.0148980

**Published:** 2016-02-16

**Authors:** Liv-Marie Eike, Brynjar Mauseth, Ketil André Camilio, Øystein Rekdal, Baldur Sveinbjørnsson

**Affiliations:** 1 Department of Molecular Inflammation Research, Institute of Medical Biology, Faculty of Health, University of Tromsø, N-9037, Tromsø, Norway; 2 Lytix Biopharma, Gaustad Alléen, Oslo, Norway; 3 Childhood Cancer Research Unit, Department of Women’s and Children’s Health, Karolinska Institute, Stockholm, Sweden; Rutgers University, UNITED STATES

## Abstract

In the present study we examined the ability of the amino acid derivative LTX-401 to induce cell death in cancer cell lines, as well as the capacity to induce regression in a murine melanoma model. Mode of action studies *in vitro* revealed lytic cell death and release of danger-associated molecular pattern molecules, preceded by massive cytoplasmic vacuolization and compromised lysosomes in treated cells. The use of a murine melanoma model demonstrated that the majority of animals treated with intratumoural injections of LTX-401 showed complete and long-lasting remission. Taken together, these results demonstrate the potential of LTX-401 as an immunotherapeutic agent for the treatment of solid tumors.

## Introduction

Host defense peptides (HDPs) are naturally occurring peptides with important antimicrobial and immunomodulatory functions, as part of the innate immune system in virtually all species of animals [1–3]. Several HDPs are reported to exhibit oncolytic properties, also termed oncolytic peptides, with an ability to cause the lysis of cancer cells [4]. This ability has been confirmed by several synthetically designed peptides [4–7]. In the present study, the effect of a cytolytic compound, LTX-401, was investigated. LTX-401 is a small amphipathic β(2,2)-amino acid-derived antitumor molecule with an amphipathic conformation. Based on structure-activity-relationship (SAR) studies on short cationic peptides (<9 amino acid residues), it was discovered that the introduction of large lipophilic groups compensated for the peptide length [8]. Additionally, by coupling two aromatic side-chains to the same carbon atom, the length could be further reduced to a minimum without losing cytotoxic activity. These discoveries led to the production and synthesis of a panel of β-peptidomimetics to help confirm the pharmacophore model of short cationic peptides [9], which dictates that such compounds should contain cationic-charged residues, in addition to bulky and lipophilic moieties for optimal activity [3, 9]. LTX-401 was chosen as the lead compound in this panel. The purpose of the present study was to investigate the cytotoxic mode of LTX-401 *in vitro*, including the ability to induce the release of danger-associated molecular pattern molecules (DAMPs), as well as the effect of LTX-401 treatment in an experimental murine B16 melanoma model.

## Material and Methods

### Reagents

**Fig 1:**
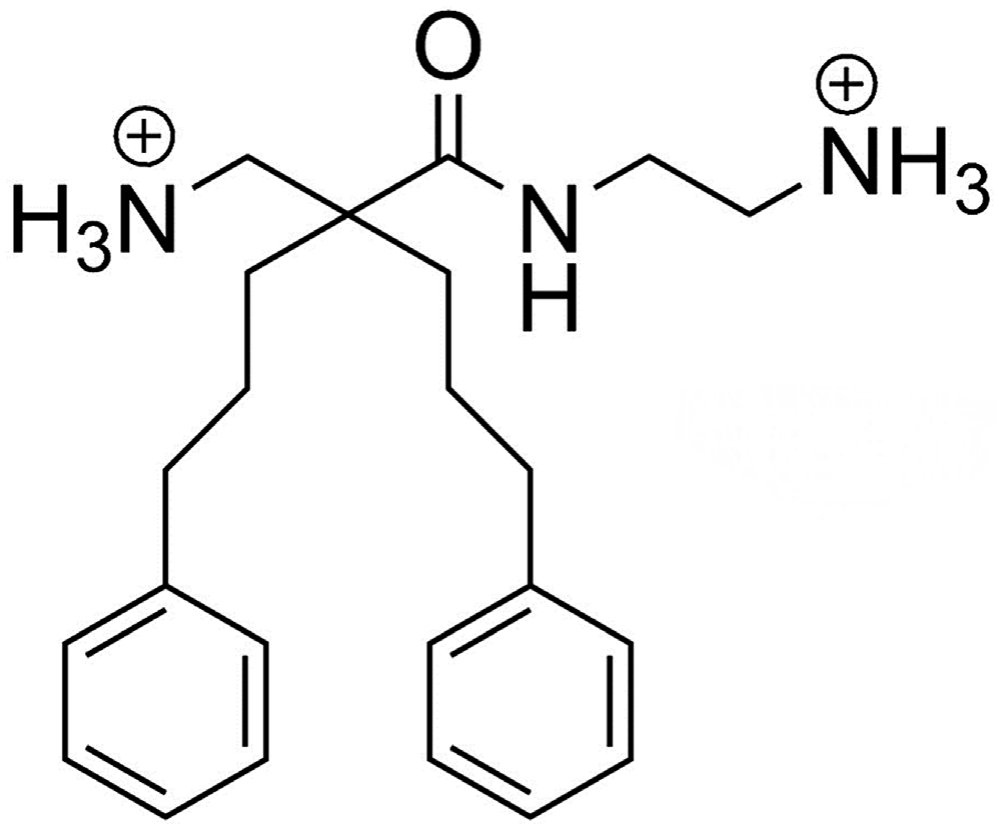
Chemical structure of the small amphipathic β(2,2)-amino acid-derived antitumor molecule LTX-401 (Mw_net_ = 367.53).

LTX-401 (Mw_net_ = 367.53) was synthetized on request by Synthetica AS (Oslo, Norway). The chemical structure is shown in Fig 1.

### Cell Cultures

JM1, a rat hepatocellular carcinoma, HEPG2 and BEL7402, both human hepatocellular carcinomas, were kindly provided by Dr. Pål-Dag Line, Director, Department of Transplantation Medicine, Oslo University Hospital. B16F1 (ATCC, CRL-6323), a murine skin malignant melanoma, MDA-MB-435S (HTB-129), a human malignant melanoma originating from a breast metastases, Malme-3M (HTB-64), a human malignant melanoma derived from a lung metastases, MRC-5 (ATCC, CCL-171), normal human lung fibroblasts, SK-N-AS (ATCC, CRL-2137), a human neuroblastoma cell line derived from a bone marrow metastases, HT-29 (ATCC, HTB-38), a human colorectal adenocarcinoma and HUV-EC-C (ATCC, CRL-1730), a human vascular endothelium cell line, were all purchased from the American Type Culture Collection (ATCC-LGC Standards, Rockville, MD, USA). HaCat, a human keratinocyte cell line, was kindly provided by Dr. Ingvild Pettersen, Department of Host Microbe Interactions, University of Tromsø. A375, a malignant melanoma cell line of human origin, was purchased from Public Health England (PHE Culture Conditions, Porton, Down, Salisbury, UK). JM1, A375, BEL7402, HEPG2 and B16F1 were all maintained in culture media consisting of DMEM (high glucose), while SK-N-AS, HT-29 and MDA-MB-435S were cultured in an RPMI-1640 medium containing 2mM L-glutamine and sodium bicarbonate. Malme-3M was cultured in RPMI-1640, containing 4 mM L-glutamine and 20% FBS (fetal bovine serum). Lastly, the three non-malignant cell lines, HUV-EC-C, MRC-5 and HaCat, were cultured, respectively, using the EGM-2 BulletKit (Lonza, MA), containing basal media and growth factors, MEM (normal glucose) also containing 2mM L-glutamine and 1% non-essential amino acids and DMEM (normal glucose) containing 2 mM L-glutamine. All media were (if not specified otherwise) supplemented with 10% FBS, with the exception of the EGM-2 BulletKit, which is delivered with an aliquot of 10 ml FBS to yield a final concentration of 2% FBS in solution.

### *In Vitro* Cytotoxicity

The MTT assay [10] was employed to investigate the *in vitro* cytotoxicity of LTX-401 against a selection of both cancer and non-malignant cell lines. Pre-cultured cells were seeded at a density between 1 x 10^4^–1.5 x 10^4^ cells/well, and the experiment was performed as previously described [5]. The results were calculated using the mean of three experiments, each with triplicate wells, and expressed as a 50% inhibitory concentration (IC_50_).

### Hemolytic Activity

The cytotoxic activity of LTX-401 against human red blood cells (RBCs) was determined by a hemolytic assay using freshly isolated blood from healthy individuals who gave their signed informed consent, and prepared as previously described [11]. RBCs were resuspended to a 10% hematocrit solution before being incubated for 1 h at 37°C with LTX-401 dissolved in PBS at concentrations ranging from 136–1358 μM (50–500 μg/ml). RBCs with PBS and 1% Triton solution alone served as a negative and positive control, respectively. After centrifuging the samples at 4,000 rpm for 5 minutes, the absorbance of the supernatant was measured at 405 nm on a spectrophotometric microliter plate reader (Thermomax Molecular Devices, NJ, USA). The protocol used for blood sampling and handling has been reviewed by the Regional Ethical Committee for Research Ethics at UiT, The Arctic University of Norway (S1 File). The protocol is in accordance with international and local human research ethical standards.

### Kill Kinetics

The killing kinetics of LTX-401 were studied against B16F1 melanoma cells, using both the 2 x IC_50_^4h^ and 4 x IC_50_^4h^ values corresponding to 54 μM and 108 μM, respectively. Cells were seeded as previously described for MTT assay, and incubated with LTX-401 solutions for 5, 15, 30, 60, 90, 120 and 240 minutes. Cells were washed once with 100 μl of serum-free RPMI-1640 after incubation, and further incubated in a 10% MTT solution (diluted in a serum-free RPMI-1640) for an additional 2 h.

### TEM Electron Microscopy

B16F1 cells were seeded in 35 mm sterile tissue culture dishes at a density of 1 x 10^4^ cells in a volume of 2 ml of culture media, and left to adhere and grow in a cell incubator at 37°C, >95% humidity and 5% CO_2_ conditions. When cultured cells reached an acceptable confluence (∼80–90%), they were incubated with the 4 x IC_50_^4h^ value of LTX-401 (108 μM) for different time points (5, 15, 30 and 60 minutes) and subsequently fixed in a PHEM buffered (0.1 M) solution containing 0.05% malachite green oxalate (Sigma), 0.5% glutaraldehyde (GA, Electron Microscopy Sciences) and 4% formaldehyde (FA, Electron Microscopy Sciences). Malachite green is more frequently being used as an additive to primary fixation in order to reduce lipid extraction commonly associated with sample processing [12, 13] and references cited [14]. Samples were immediately loaded and processed *en bloc* using the PELCO Biowave Pro Laboratory Microwave (Ted Pella, Inc.), a newly introduced method considered to be advantageous over conventional bench protocols, since it reduces sample preparation from days to hours. For all microwave steps, samples were microwaved at 23°C with a 50°C cut-out temperature. Both malachite green/GA/FA and osmium-reduced ferrocyanide (0.8% K_3_Fe(CN)_6_/1% OsO_4_) fixation steps were run in “power on/off” cycles of 2 minutes on, 2 minutes off (100 W), with vacuum. Samples were rinsed four times with 0.1 M PHEM buffer between each step (two bench rinses and two final rinses for 40 seconds at 250 W) before being stained with 1% tannic acid (Electron Microscopy Sciences, PA, USA) and 1% aqueous uranyl acetate (Electron Microscopy Sciences, PA, USA) under “power on/off” cycles of 1 minute on, 1 minute off (150 W), with vacuum. Samples were rinsed as previously described between each staining procedure, in addition to being microwaved twice in water at 250 W (vacuum off). The dehydration of samples occurred through a graded ethanol series (25%, 50%, 70%, 90% and 100%), microwaving at 250 W for 40 seconds on each grade (vacuum off) before being immersed *en bloc* with a 50% Epon resin (AGAR, DDSA, MNA and DNP-30), and increased to 100% for the two final infiltration steps, 3 minutes each at 250 W (with vacuum cycle; 20 seconds on, 20 seconds off). Samples were then polymerized overnight at 60°C. Ultrathin sections (70 nm) were prepared and placed onto standard formvar, carbon-stabilized copper grids. Next, samples were examined on a JEOL JEM-1010 transmission electron microscope, and images were taken with an Olympus Morada side-mounted TEM CCD camera (Olympus soft imaging solutions, GmbH, Germany).

### Release of High Mobility Group Box 1 (HMGB1)

B16F1 cells were seeded at a density of 2 x105cells/well in 6-well plates in a complete medium, and allowed to adhere overnight. Cells were treated with 108 μM of LTX-401 (4 x IC_50_^4h^), and incubated at 37°C (>95% humidity and 5% CO_2_) for different time points (10, 30, 60, 90 and 120 minutes). Serum-free RPMI 1640 was used as a negative control, and supernatants (S) were collected and centrifuged at 1,400g for 5 min before being up-concentrated using Amicon Ultra-0.5 Centrifugal Filter units with Ultracel-50 membrane (Milipore, Norway). Cell lysates (L) were harvested after washing with 2 mL of serum-free RPMI-1640 and subjected to a lysis buffer (mastermix) containing 2x NuPAGE LDS Sample buffer (Invitrogen, Norway), a 1x NuPAGE Sample Reducing Agent (Invitrogen, Norway) and 50% sterile H_2_0. Both supernatants and lysates were boiled at 70°C for 10 min and resolved on NuPAGE Novex 4–12% Bis-Tris Gels (Millipore, Norway) before being electrotransferred to a polyvindiline difluoride (PVDF) membrane (Millipore, Norway). Membranes were blocked for 1 h using 5% non-fat dry milk in TBS-T and next hybridized with HMGB1 antibody (rabbit polyclonal to HMGB1 –ChIP Grade, Abcam, UK) overnight at 4°C, followed by horseradish peroxidase- (HRP) conjugated secondary antibody (goat polyclonal anti-rabbit IgG, Abcam, UK) for an additional 2 h at room temperature before being developed using WB Luminol Reagent (Santa Cruz Biotechnology, Heidelberg, Germany), and imaged/scanned using ImageQuant LAS 4000 and ImageQuant software (GE Healthcare).

### Release of Cytochrome *c*

B16F1 cells were plated onto 96-well culture plates as previously described above for the MTT assay. Cells were treated with 108 μM (4 x IC_50_^4h^) for designated time points (30, 60, 90, 120 and 240 minutes), while control cells were preserved in serum-free RPMI-1640 only until the experimental endpoint. Supernatants were collected and diluted 1/5 in a serum-free RPMI 1640 before being evaluated using a Rat/Mouse Cytochrome *c* Quantikine ELISA kit (R&D Systems, USA), according to the instructions of the manufacturer. The concentration of cytochrome *c* was calculated by interpolation values on the provided standard curve.

### Release of ATP

B16F1 cells were seeded as previously described for the MTT assay, and treated with the 2 x IC_50_^4h^ value of LTX-401 (54 μM) for different time points (10, 30, 60, 90 and 120 minutes). Serum-free RPMI 1640 treated cells functioned as a negative and blank control, respectively. Extracellular levels of ATP were measured at the end of the experiment using a luciferin-based ENLITEN ATP Assay kit (Promega, Madison, WI, USA), in which ATP-driven chemoluminescence was recorded on a Luminescence Microplate Reader (Labsystems Luminoskan^®^, Finland), and expressed as relative light units (RLU).

### LysoTracker

#### Flow cytometry

B16F1 cells were seeded in 6-well plates and allowed to adhere overnight. Cells were then treated with the 1 x IC_50_^4h^ of LTX-401 (27 μM) for 60 minutes, with 40 nM LysoTracker DND-26 (Invitrogen) added in the last 5 minutes. Untreated cells were used as a control. Cells were trypsinized and investigated on a FACS Calibur Flow cytometer, and the results were processed using FlowJo Software (Tree Star, Inc., Ashland, OR, USA). PI was utilized in some experiments to gate away cells with compromised plasma membrane.

#### Confocal microscopy

B16F1 cells were seeded in 8-well plates (Nunc) at 1 x 10^4^ cells/well and allowed to adhere overnight. Next, cells were treated with 27 μM of LTX-401 for 60 minutes and labelled with LysoTracker DND-26 for 5 minutes and with Hoecsht33342 for nuclear staining. Cells were immediately investigated on a Zeiss Confocal microscope with a controlled temperature and atmosphere.

### Animals

Female C57BL/6 wild-type mice, 5–6 weeks old, were obtained from Charles River, United Kingdom. All animals were housed in the same room and in cages (containing 2–5 mice) specially designed for mice, with a 12h/12h day-night cycle, and allowed *ad libitum* access to high quality food and water. Each cage contained environmental enrichments such as nest material, chewing sticks and housing. Animals were weighed weekly and monitored several times per week, and no unexpected deaths were observed. All experiments were approved by the Norwegian National Animal Research Authority (NARA), and conducted in accordance with local and European Ethical Committee guidelines, NARA approval ID: 6586.

### Tumor Treatment

**Fig 2:**
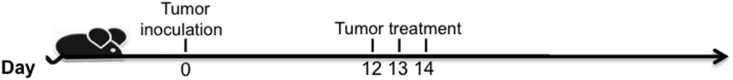
Tumor-treatment schedule.

B16F1 cells were harvested, washed in serum-free RPMI and injected intradermally (i.d) into the right side of the abdomen in C57BL/6 mice (7.5 x 10^4^ B16F1 cells per mouse/50 μl RPMI-1640). Palpable tumors (20–30 mm^2^) were injected intratumorally (i.t) with either LTX-401 or a vehicle (saline) once per day for three consecutive days (Fig 2). Animals were euthanized according to humane endpoints such as a weight loss of >10%, general physical discomfort or reduced activity, severe tumor necrosis or ulceration, or if the tumor size exceeded 130 mm^2^. All animals were euthanized using cervical dislocation.

### Histological Examination

Tumors were fixated by perfusion using ZN^2+^ fixative. After fixation and paraffin embedding, sections were deparaffinized using xylene and graded alcohol before hydration and washing with PBS. Antigen retrieval was performed in a sodium citrate buffer (pH 6) in a microwave oven, and endogenous peroxidase was subsequently blocked by 0.3% H_2_O_2._ Sections were incubated overnight at 4°C with a primary antibody (rabbit anti-CD3; Dako). An anti-rabbit-horseradish peroxidase SuperPicTure Polymer detection kit (Invitrogen, San Francisco, CA, USA) was used as a secondary antibody. A matched isotype control was used as a control for non-specific background staining, and a routine standard staining was performed with hematoxylin and eosin.

### Statistical Analysis

Results are presented as a mean ± standard error of mean (SEM) or standard deviation (SD) of at least two independent experiments. MTT assays were conducted twice with three parallels and cytochrome *c* assays were conducted twice with two parallels, while ATP assays were conducted three times with two parallels. Cytochrome *c* release- and ATP release data were compared using one-way ANOVA and a multiple comparison test, and we considered a P-value ≤0.05 to be considered statistically significant. Animal survival plots were tested for significance using a Mantel-Cox test.

## Results

### LTX-401 Rapidly Induces Cell Death

In this study, LTX-401 effectively reduced the viability of several tumor cell lines *in vitro*, with a similar degree of cytotoxicity against non-malignant cell lines such as HUV-EC-C endothelial cells, HaCat keratinocytes and MRC-5 fibroblasts (Table 1). LTX-401 displayed the highest cytotoxic activity against the human malignant melanoma cell line MDA-MB-435S (13.5 μM), and was least active against the human hepatocellular carcinoma cell line HEPG2 (35.4 μM). For the remaining cell lines, LTX-401 exhibited similar IC_50_ values, varying slightly within the range of 19–32 μM. No *in vitro* hemolytic activity against RBCs was observed using the same concentrations required for the induction of cell death in cancer cell lines. A 50% hemolysis was observed using higher concentrations of LTX-401 (400 μg/ml = 1087 μM) (Table 1).

**Table 1:** LTX-401 is active against a range of different cancer cells, but exhibits a low hemolytic activity. Data from three or more experiments are presented as mean ±SD. IC50 is the inhibitory concentration, resulting in a killing of 50% of the cells.

Cell lines	LTX-401 IC_50_^a^ ± SD (μM)
JM1 (Rat hepatocellular carcinoma)	22.8 ± 3.3
HEPG2 (Human hepatocellular carcinoma)	35.4 ± 0.6
BEL7402 (Human hepatocellular carcinoma)	26.7 ± 0.4
B16F1 (Murine skin malignant melanoma)	23.3 ± 3.9
MDA-MB-435S (Human malignant melanoma)	13.5 ± 1.4
Malme-3M (Human malignant melanoma)	19.3 ± 0.4
HT-29 (Human colorectal adenocarcinoma)	31.7 ± 2.9
A375 (Human skin malignant carcinoma)	30 ± 0.9
SK-N-AS (Human neuroblastoma)	30.6 ± 0
MRC-5 (Human lung fibroblasts)	22.9 ± 1.4
HUV-EC-C (Human vascular endothelium)	18.4 ± 1.9
HaCat (Human keratinocytes)	22 ± 2.2
RBC (Human)	>1087

^**a**^ The peptide concentration killing 50% of the cells

Kinetic experiments were performed to determine the time course of the cytotoxic activity of LTX-401 against B16 melanoma cells. Two different concentrations representing the 2 x IC_50_^4h^ and 4 x IC_50_^4h^ of LTX-401, i.e. 54 μM and 108 μM, respectively, were used to assess the dose-dependent effect. As revealed by initial pilot studies, these two concentrations were also employed for the majority of *in vitro* experiments, and in particular when studying the release of DAMPs, as lower concentrations failed to induce such a modality.

**Fig 3:**
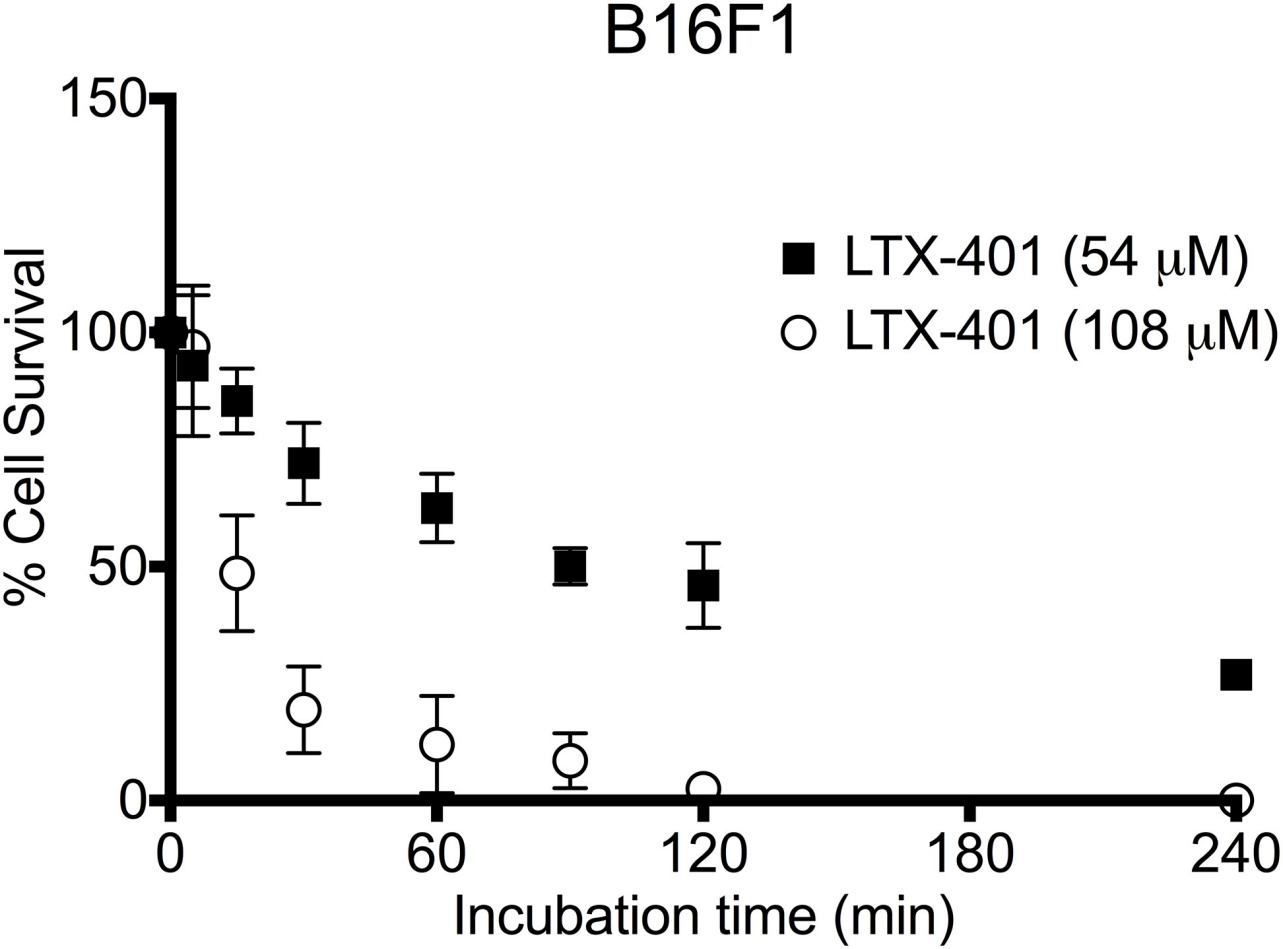
LTX-401 rapidly induces cell death in B16F1 melanoma cells. The determination of cell viability after a short incubation at two different concentrations of LTX-401 was analyzed after designated time points (5, 15, 30, 60, 90, 120 and 240 minutes), which revealed a decreased viability after 60 minutes of incubation. Data represent three experiments performed in triplicate presented for each time point as mean ± SEM.

LTX-401 (108 μM) exhibited rapid killing kinetics, with a kill ratio of 50% after 15 min, followed by nearly 100% cell death two hours after start of treatment. In contrast, cells incubated with 54 μM of LTX-401 followed a gradual inhibition of cell viability, with a 50% cell survival after two hours (Fig 3).

### LTX-401 Treatment Causes Ultrastructural Changes in Melanoma Cells

**Fig 4:**
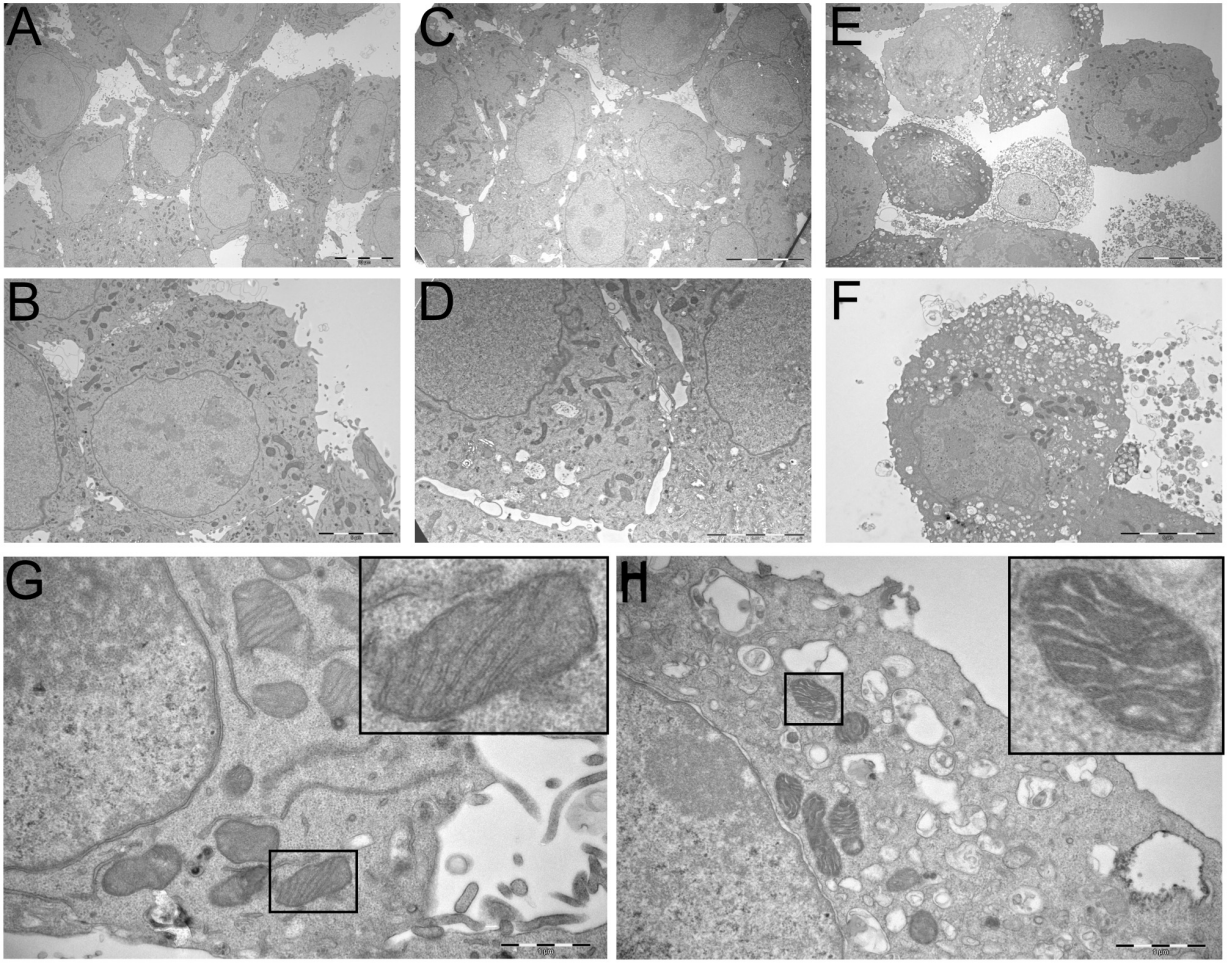
LTX-401 treatment induced ultrastructural changes with vacuolization. Representative TEM micrographs of B16F1 cells treated with LTX-401 (108 μM). Untreated control cells (A and B) were kept in a serum-free RPMI 1640 only until the experimental endpoint (60 minutes), and compared with cells treated for both 5 min (C and D) and 60 min (E and F); scale bars = 10 μm for A, C, E, 5 μm for B, D, F.

To further investigate the mode of action underlying the cytotoxic activity of LTX-401, B16F1 cells were incubated with the 4 x IC_50_^4h^ of LTX-401 (108 μM) for 5 and 60 min, respectively. Untreated cells served as a control, and were incubated in a serum-free RPMI 1640 until the experimental endpoint (60 min). After incubation, all cells were fixed and prepared for TEM studies. TEM images of untreated B16F1 cells revealed a rough surface characterized by frequent microvillus-like protrusions on the plasma membrane (Fig 4A and 4B). The cytoplasm consisted of several electron-dense mitochondria, and visibly smooth ER and Golgi apparatus (Fig 4A and 4B). The majority of LTX-401 treated cells lost their surface morphology after 5 min, and the majority of cells were without significant alterations except for a slight vacuolization of the cytoplasm (Fig 4C). After 60 minutes of incubation, treated cultures demonstrated a heterogeneous population of cells, including both heavily vacuolated and large non-vacuolated cells (Fig 4E). Large portions of cells were necrotic, as was clearly seen by the loss of plasma membrane integrity and leakage of cell constituents (Fig 4E and 4F). The chromatin structure was more or less unaffected by LTX-401 treatment, albeit slightly condensed in a number of cells. Furthermore, a higher magnification revealed no significant ultrastructural changes in the mitochondria of the majority of LTX-401 treated cells (Fig 4H) compared to controls (Fig 4G). Cells treated for 60 minutes (Fig 4H) displayed intact inner and outer mitochondrial membranes, albeit with some degree of mitochondrial swelling. Overall, these observations demonstrate that LTX-401 kills by a lytic mode of action, accompanied by cellular swelling, mitochondrial swelling and vacuolization of the cytoplasm.

### LTX-401 Treatment Results in the Release of DAMPs in Melanoma Cells

**Fig 5:**
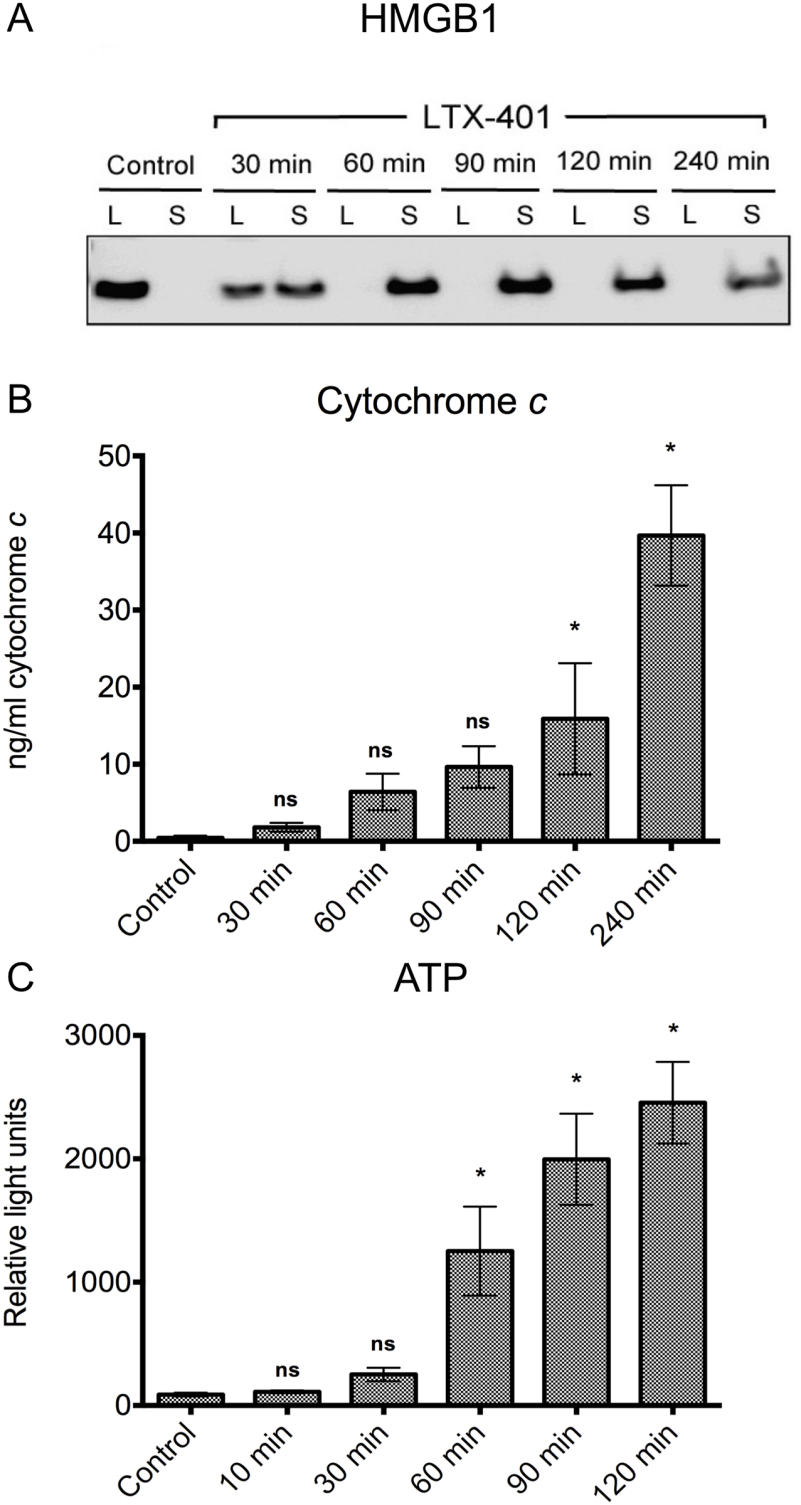
LTX-401 induces the release of danger signals. (A) Release of HMGB1 into the supernatant of LTX-401-treated cells as determined with Western blot. Translocation of the nuclear protein HMGB1 from the cell lysate (L) to the culture supernatant (S) was evident after 30 minutes of treatment with LTX-401 (108 μM), and the translocation was absolute after 90 minutes of incubation. Control cells showed no translocation after 240 minutes. Experiment was conducted thrice, and this figure is a digital image of a representative blot; (B) B16F1 cells were treated with LTX-401 (108 μM) for different time points (30, 60, 90, 120 and 240 minutes) before determining the amount of cytochrome *c* in supernatants using an ELISA assay. The results are presented as mean +/- SEM; (C) B16F1 cells were treated with LTX-401 (54 μM) for designated time points (10, 30, 60, 90 and 120 min). The quantification of ATP level was performed by luciferase bioluminescence, and the results are presented as mean +/- SEM.

Next, we wanted to characterize the ability of LTX-401 to induce the release of DAMPs. HMGB1 is a non-histone nuclear protein, and when released extracellularly it can act as a DAMP by binding to toll-like receptors (TLRs) or receptor for advanced glycation end-products (RAGE) [15]. The release of HMGB1 from B16F1 cells into cell culture supernatants was assessed by Western blot analysis. The translocation of HMGB1 was detected, with the release of HMGB1 occurring after 30 min of treatment. Non-treated control cells displayed no release of HMGB1 into the supernatant, as seen by the complete detainment of the protein within the lysates (Fig 5A).

Cytochrome *c* is considered a mitochondrial-derived DAMP [16], and extracellular cytochrome *c* has been reported to induce NF-kB activation and the release of proinflammatory cytokines [17]. To study whether LTX-401 was able to induce the release of cytochrome *c* from LTX-401-treated B16F1 cells, an ELISA assay was employed to measure the amounts of cytochrome *c* in the cell culture medium after treatment. A quantitative analysis demonstrated the presence of cytochrome *c* in the supernatant following LTX-401 treatment with the 4 x IC_50_^4h^ value of LTX-401 (108 μM) (Fig 5B) after 120 min of treatment. The release of cytochrome *c* followed a gradual increase over time, reaching a peak of approximately 40 ng/ml at the experimental endpoint (Fig 5B).

ATP is reported to have immunogenic properties when released from dying and/or stressed cells, including cancer cells succumbing to conventional chemotherapy [18, 19]. To investigate whether LTX-401 was able to induce the release of ATP from B16F1 cells, a luciferin-luciferase-based reaction assay was employed. The extracellular concentration of ATP quickly rose 60 min after initiating treatment with a gradual increase towards 120 min (Fig 5C).

### Treatment with LTX-401-Induced Loss of Lysosomal Integrity in Melanoma Cells

**Fig 6:**
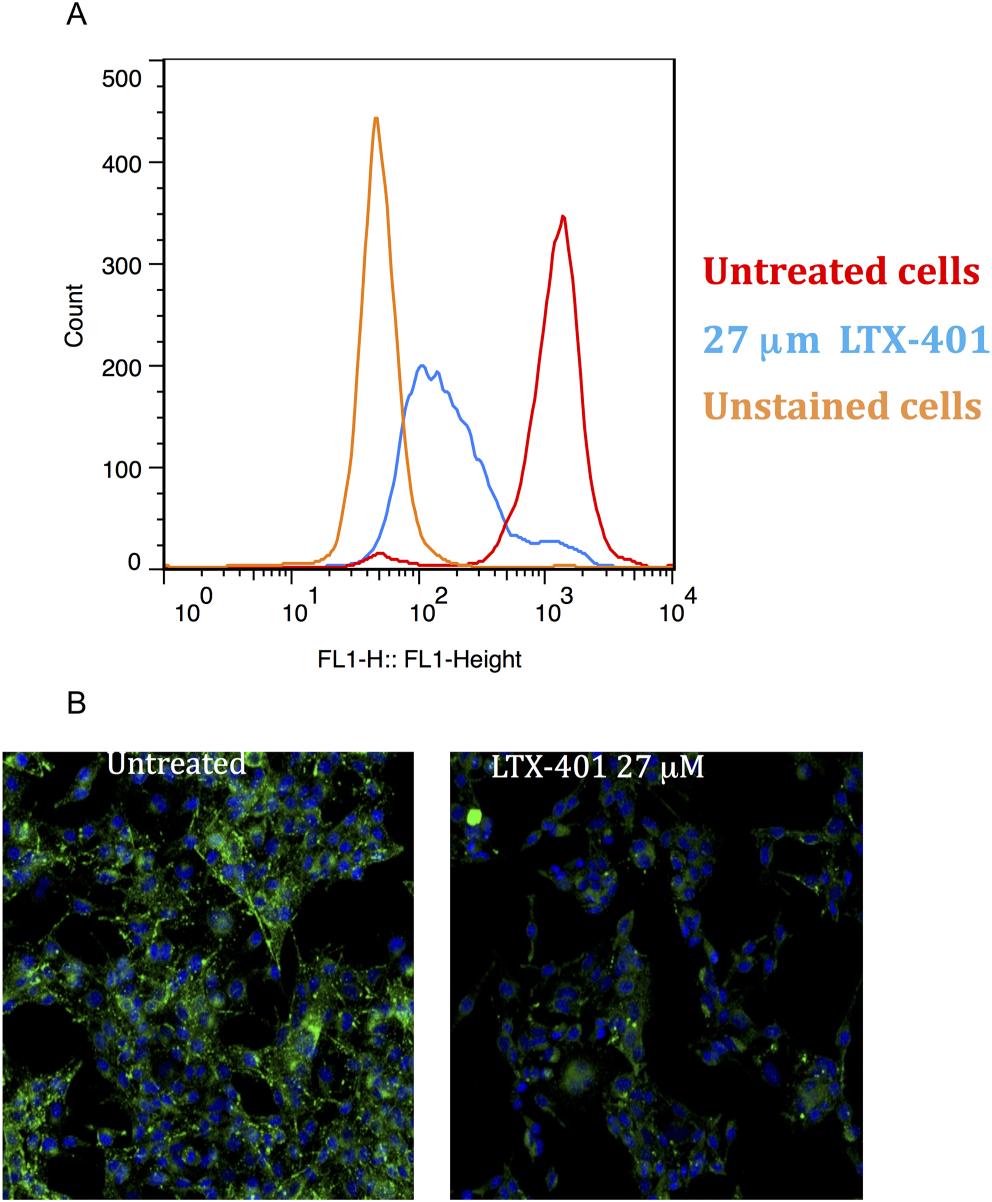
LTX-401 treatment induced loss of lysosomal integrity. (A) B16F1 cells were treated with LTX-401 (27 μM) for 60 minutes before determining the signal strength from LysoTracker (DND-26) using flow cytometry. The figure is representative for one of three conducted experiments; (B) B16F1 cells were treated with LTX-401 (27 μM) for 60 minutes, and the signal from LysoTracker was imaged using confocal microscopy (z-stack, maximum intensity projection).

Next, we wanted to investigate whether LTX-401 caused any changes in lysosomal integrity. Treatment with LTX-401 resulted in a loss of signal from the acidophilic dye LysoTracker DND-26, as shown with flow cytometry and confocal microscopy. This dye accumulates in acidic organelles such as lysosomes and melanosomes. The effect was shown not to be cell-type specific, as similar results were also obtained in lymphoma cells (data not shown). As demonstrated by both flow cytometry and confocal microscopy, 27 μM of LTX-401 induced a decreased signal in B16F1 melanoma cells after 60 minutes of incubation (Fig 6A and 6B).

### Intratumoral Injection of LTX-401 Leads to Complete Regression of B16F1 Murine Melanoma

**Fig 7:**
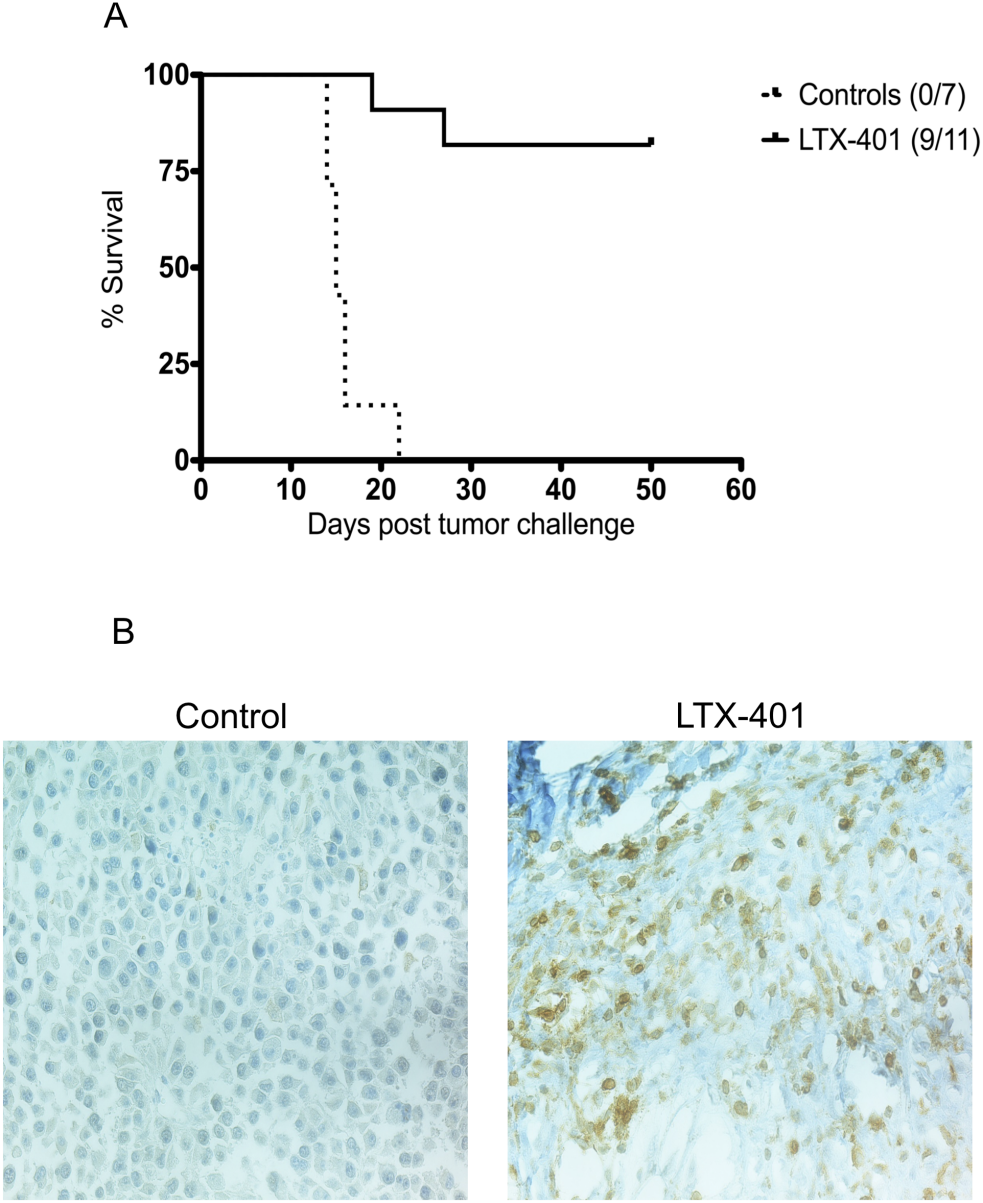
Intratumoral treatment induced complete regression in B16F1 melanomas. (A) The intratumoral treatment of murine melanoma induced a rapid and complete regression in 9/11 animals, and offered long-term protection against a rechallenge in the majority of the cured mice. Palpable B16F1 tumors were injected with either a sterile 0.9% NaCl (vehicle controls) or 5 mg/ml LTX-401 once a day for three consecutive days. P<0.0001 (Mantel-Cox test); (B) Infiltrating CD3+ lymphocytes were detected in tumors following one single injection of LTX-401 (7 days post-injection) in a murine experimental animal model.

To assess the antitumor effects of LTX-401 *in vivo*, palpable B16 melanomas were injected i.t with LTX-401 (or vehicle only) once a day for three consecutive days. The majority (9/11) of the animals demonstrated a complete and lasting tumor regression after intratumoral treatment with LTX-401 (Fig 7A).

To study the underlying mechanisms behind tumor regression, tumors were dissected for histological analysis. Samples were collected from selected animals in the treatment group and control group on days 2 and 7 post-treatment with a single i.t injection. The immunolabelling of tumor tissue with anti-CD3 antibody revealed that a majority of the infiltrating cells were CD3^+^ T cells, whereas tumors injected with vehicle only exhibited tumors with a viable tumor tissue, with minimal necrosis and few lymphocytes (Fig 7B)

## Discussion

The ability of cancer cells to evade immunosurveillance has recently been acknowledged as an emerging hallmark of cancer [20]. Immunogenic cell death (ICD) is defined as the release of immune-potentiating molecules termed danger-associated molecular pattern molecules (DAMPs), and thus is critical in establishing an immune response against cancer cells [21]. Due to the ability of some cytostatic compounds to induce ICD [18, 22], it has also been shown that the immune system plays an important role in cancer eradication as a response to conventional treatment. ICD has additionally been suggested as a determinant for the long-term success of anticancer therapies [21]. Oncolytic therapy is a new and promising therapeutic approach against solid tumors, where cancer cells are lysed *in situ* with the subsequent release of DAMPs [5, 23–25].

The recently designed amino acid derivative LTX-401 has been reported to exhibit anticancer activity [26]. In the present study, we demonstrate that LTX-401 exerts anticancer activity against a range of cancer cell lines, including B16 melanoma (Table 1). However, LTX-401 is also active against normal endothelial cells, keratinocytes and fibroblasts, but displays a low hemolytic activity (Table 1). We have previously designed shorter peptides with the potential to adopt an α-helical coil structure based on structure-activity relationship studies (SAR) on bovine lactoferricin (LfcinB) derivatives. These peptides have been shown to kill cancer cells more effectively than the naturally occurring LfcinB (25-mer), both *in vitro* and *in vivo* [5, 23]. SAR studies revealed that the size of the aromatic sector, and hence the overall higher hydrophobicity, is an important factor that will potentiate the anticancer activity of the peptide, but it could also increase the efficacy against untransformed cells (Fig 1) [27, 28]. This is supported by recent observations, in which anticancer peptides with greater hydrophobicity were less selective and displayed more cytotoxicity against normal cells [29, 30]. Such a structural parameter may explain in part the lack of selectivity, and hence an unspecific targeting of also non-malignant cells by LTX-401, as the two aromatic side chains give it a high hydrophobicity, thereby making it more prone to interact with phospholipid bilayers. Furthermore, it is likely that the low positive charge of LTX-401 (+2) reduces its capacity of binding strongly to anionic surface components on cancer cells via electrostatic forces. It is plausible that a higher cationicity would therefore render non-malignant cells less susceptible to killing, but since LTX-401 is proposed to be used in the local treatment of solid tumors, the selectivity against cancer cells is less important, in contrast to anticancer drugs designed for systemic use.

Kinetic experiments were conducted in order to study the impact of different concentrations of LTX-401 against B16F1 melanoma cells over time. The concentrations used in the kinetic studies represented the 2 x IC_50_^4h^ and 4 x IC_50_^4h^ values. The cellular survival was reduced to a minimum after 90–120 min of using the 4 x IC_50_^4h^ value (108 μM) (Fig 3). By contrast, commonly used chemotherapeutic agents require longer incubation periods to exert a significant anticancer effect [6].

To study the morphological changes of LTX-401 treated cancer cells, transmission electron microscopy (TEM) studies were performed. These studies revealed an early loss of surface morphology and a slight vacuolization of the cytoplasm of treated B16F1 cells (Fig 4C and 4D). An increase in cell size was also evident shortly after the start of LTX-401 treatment. By prolonging the incubation period of treated cells (to 60 min), TEM images demonstrated an increased vacuolization, and revealed that cells were killed by a lytic mode of action that resulted in a loss of cell membrane integrity and the subsequent leakage of intracellular contents into the extracellular milieu (Fig 4E and 4F).

The formation of vacuoles containing cytoplasmic material may constitute a transitional state in which cells respond to acute intracellular stress exerted by LTX-401 on different organelles. The mitochondria displayed normal morphology five minutes post treatment, while showing evidence of swelling at experimental endpoint (60 min). In all the samples, the cells displayed a heterogeneous morphology, and we observed differences between individual cells among healthy control cells. Cell lines derived from the B16 cell line are known to consist of a heterogeneous population of both spindle-shaped and epithelial-like cells [31, 32]. These different phenotypes could possibly respond differently to treatment with anticancer substances, including LTX-401, which may partially explain the heterogeneous morphology of the treated cells.

When the concept of immunogenic cell death (ICD) was introduced, it was recognized that the mode of cancer cell death plays an important role in determining the outcome and success of selected anticancer therapies, including radiotherapy and several commonly used chemotherapeutic regimens [18, 33, 34]. The activation of potent anti-tumor immune responses has been shown to rely on a series of cellular and biochemical events culminating in the release of DAMPs from dying and/or stressed tumor cells, including surface-exposed calreticulin (CRT), secreted adenosine triphosphate (ATP) and passively released High Mobility Group Box-1 protein (HMGB1). These three molecules are considered the hallmarks of ICD [21, 35]. When interacting with their respective receptors, DAMPs, along with tumor antigens, may orchestrate the recruitment and activation of dendritic cells (DCs) into the tumor bed, which may later home to draining lymph nodes to active tumor-specific CD8^+^ T cells [15, 36, 37]. In the present study, B16F1 melanoma cells treated with LTX-401 *in vitro* were screened for the release of ATP and HMGB1 using a luciferase assay and Western blot analysis, respectively. These experiments revealed that LTX-401 treatment induced the release of HMGB1 and ATP in B16F1 cells (Fig 5A and 5C). The translocation of HMGB1 from the intracellular compartment into the supernatant was evident in B16F1 cells treated with LTX-401. The extracellular release of HMGB1 from post-apoptotic and/or necrotic cells is capable of sustaining and augmenting an anti-tumorigenic environment by the attraction of inflammatory leukocytes and the stimulation of pro-inflammatory cytokines such as TNF-α and IL-6 [38, 39]. In addition, HMGB1 serves a pivotal role in the maturation of DCs and favors both processing and the presentation of tumor antigens to naïve T cells, thereby establishing a link between innate and adaptive anti-tumor immune responses [33, 40, 41]. However, the release of HMGB1 from dying cancer cells is not solely consistent with the establishment of anti-tumor immune responses as redox modifications induced by the cell or in the extracellular milieu, which may alter and even attenuate its DAMP activities [42, 43].

When an ATP is released or secreted into the extracellular milieu by tumor cells, it acts on purinergic receptors to help facilitate the recruitment of immune cells into the tumor bed [44]. Moreover, when binding to P_2_X_7_ receptors on DCs, ATP stimulates the assembly of NLRP3 inflammasome, and initiates a series of downstream events that ultimately result in the production and release of IL-1β, a cytokine required for the priming of IFN-γ producing tumor-specific CD8^+^ T cells [45–47]. ATP was released from B16F1 in an increasing manner during the experimental time period.

Mitochondrial DAMPs (mtDAMPs) include ATP, mitochondrial DNA, formyl peptides, oxidized cardiolipin and cytochrome *c* [16]. These molecules are considered to be potent immune activators, as mitochondria bears a striking resemblance to bacteria [16]. Cytochrome *c* marks one of the early events during apoptotic cell death, in which its release from the mitochondrial intermembrane space into the cytosol controls the assembly of the apoptosome and activation of procaspase-9, thus acting like an intracellular danger signal [48]. The release of cytochrome *c* is also reported to occur from cells succumbing to necrosis [49]. Furthermore, extracellular cytochrome *c* induces the activation of NF-kB and the release of other proinflammatory cytokines and chemokines [17]. Elevated serum levels of cytochrome *c* have been observed in SIRS patients and are linked to poor survival [50]. A cytochrome *c* ELISA assay was performed to help assess the capability of LTX-401 to release mtDAMPs. Treatment with LTX-401 was shown to induce the extracellular release of cytochrome *c* from B16F1 melanoma cells *in vitro* (Fig 5B). Interestingly, while TEM micrographs confirmed the lysis of B16F1 cells after 60 min, the release of cytochrome *c* was not significantly different from controls until after 120 min, which could indicate that the mitochondria is still somewhat intact and continues to retain its cytochrome *c* for a while after cell lysis.

The electron microscopy studies support the notion that mitochondria in B16F1 cells are not initially affected by LTX-401 treatment, and the timing of cytochrome *c* release could imply that the lysis of the mitochondria is secondary to the lysis of the cell. Previous mode of action studies by Ausbaucher et al. supports these findings, in which LTX-401 did not compromise the mitochondrial membrane potential as measured by TMRE [51].

However, LTX-401 is a small molecule with an amphipathic structure, thus possessing the potential to bypass the plasma membrane, subsequently targeting intracellular structures. We therefore wanted to investigate other potential intracellular targets involved. The effect of LTX-401 treatment on acidic organelles was assessed by the use of lysosomal dye LysoTracker DND-26, with the confocal imaging of live cells demonstrating that LTX-401 treatment significantly altered the fluorescence of the acidophilic dye LysoTracker, even before gross morphological changes occurred. This observation was confirmed using flow cytometry analysis with the same concentrations and incubation time (Fig 6). The loss of fluorescence indicates that acidic organelles in the cells are compromised due to treatment. Even so, B16F1 cells also harbor melanosomes, which are acidic lysosome-related organelles. Consequently, we repeated the experiment in a non-melanocytic cell line (A20, murine lymphoma), and achieved similar results (data not shown). These findings suggest that the lysosomes are among the intracellular targets of LTX-401.

To help assess the anticancer effects of LTX-401 against highly aggressive and poorly immunogenic B16 melanomas, established tumors were treated by intratumoral injections of LTX-401 (5 mg/ml) once a day for three consecutive days. The majority of treated animals displayed a complete regression of intradermally established tumors (Fig 7A). Histological examinations of tumors treated with LTX-401 revealed an increased infiltration of CD3^+^ T cells (Fig 7B). This indicates that the cytolytic effect exerted by LTX-401 provides a favorable immunogenic environment *in situ*, thus resulting in the infiltration of lymphocytes.

Immunotherapeutic strategies aim to mount a specific T-cell response against tumor cells. Intratumoral immunotherapy for melanoma is a promising approach, with several preclinical and clinical trials reporting exciting results [25, 52–57]. In our study, we have investigated the anticancer efficacy and mode of action of a small lytic amino-acid derivative LTX-401. Melanoma cells treated with LTX-401 demonstrated features of immunogenic cell death, as shown by the release of DAMPs such as ATP, HMGB1 and cytochrome *c*. Furthermore, LTX-401 induced complete regression of highly aggressive and poorly immunogenic murine B16 melanomas. In conclusion, our results demonstrate the potential of LTX-401 as a promising immunotherapeutic agent.

## Supporting Information

S1 File Ethical statement blood sampling. (PDF)
