# Supplementary material for: The Cytolytic Amphipathic β(2,2)-Amino Acid LTX-401 Induces DAMP Release in Melanoma Cells and Causes Complete Regression of B16 Melanoma
Source: PLoS One. 2016 Feb 16;11(2):e0148980. doi: 10.1371/journal.pone.0148980 (PMC4755540; doi:10.1371/journal.pone.0148980)
Supplement: S1 File — (PDF) [file pone.0148980.s001.PDF]

UiT
THE ARCTIC UNIVERSITY OF NORWAY

The Faculty of Health Sciences/
Department of Medical Biology
Your reference.:
Our reference.:
Date: December 17, 2015

To whom it may concern

Formal Statement of Ethical Practice

Concerning ethical practice of blood sampling; Manuscript Plos One (Pone-D-15-45349), "The cytolytic amphiphatic b(2,2) amino acid LTX-401 induces DAMP-release in melanoma cells and causes complete regression in B16 melanomas" by Eike, L.M. et al.

The protocol used for blood sampling and handling has been reviewed by the Regional (Northern Norway) Ethical Commitee for Research Ethics at UiT, The Artic University of Norway. The Protocol is in accordance with international and local human research ethical standards.

Sincerely,

Siv Rist Richardsen

Siv Rist Richardsen
Head of administration

siv.r.richardsen@uit.no
+47 776 44604

UNIVERSITY OF TROMSØ
